# Repurposing a Mature Fluvial Sandstone Reservoir in Colombia for Miscible CO_2_‑EOR and Storage: Scheme Optimization and Efficiency–Retention Trade-Offs

**DOI:** 10.1021/acsomega.6c00107

**Published:** 2026-05-12

**Authors:** Imam Abed, Rubén H. Castro, Gabriel Restrepo, Prashant Jadhawar

**Affiliations:** † School of Engineering, 1019 University of Aberdeen, Aberdeen AB24 3UE, Scotland, U.K.; ‡ Sierracol Energy, Torre Once 93, Calle 93A # 10-54, Bogotá 110121, Colombia; § Rock Flow Dynamics, Bogotá 110111, Colombia

## Abstract

This study evaluates CO_2_-enhanced oil recovery with associated storage in a mature fluvial sandstone reservoir in Colombia under ongoing waterflood. A compositional sector model was built in tNavigator using a Peng–Robinson equation of state tuned to laboratory data. Minimum miscibility pressure for a flare-gas-derived injectant containing 50.74 mol % CO_2_ was computed via the multiple-mixing-cell method, confirming miscibility within the operating pressure envelope. Forecasts were used to compare continuous (CGI), alternating (WAG), tapered, and hybrid schemes to improve sweep conformance. Results show that all CO_2_ strategies increased cumulative oil (*Np*) and recovery factor (*RF*) relative to continued waterflood, with incremental *RF* of 5.6–12.0%. The hybrid CGI-WAG delivered the largest oil uplift, with the preferred variant achieving a CO_2_ utilization of 72 kg CO_2_/stb-inc and net storage of 13.3 kt CO_2_. Tapered WAG maximized storage (23.5 kt CO_2_) but at higher utilization (268 kg CO_2_/stb-inc), consistent with a larger water fraction and partial gas placement below the water–oil contact. These outcomes quantify the efficiency–retention trade-off and demonstrate a viable route to repurpose flare gas for recovery and storage. Implementation of CO_2_-compatible materials, corrosion inhibition, and surveillance is addressed to ensure mechanical integrity.

## Graphical abstract

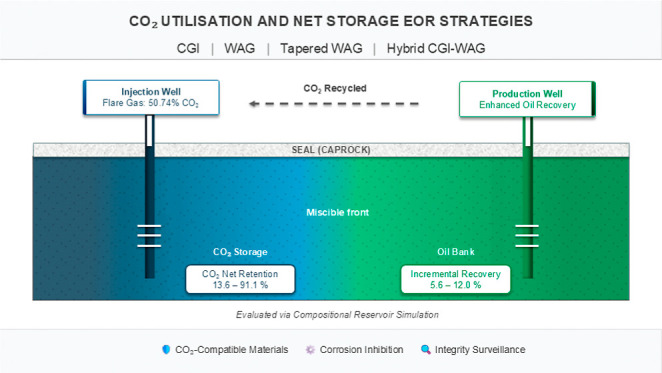

## 1 Introduction

Carbon dioxide (CO_2_) injection has been used to mobilize residual oil through the mechanisms of swelling, viscosity reduction, extraction, and vaporization of intermediates, reduction of interfacial tension (up to zero), followed by the suppression of capillary pressure when pressure exceeds the minimum miscibility pressure (MMP), thus enhancing microscopic oil displacement.^1^ Under immiscible conditions, swelling and viscosity reduction still provide uplift, but sweep can be limited without a mobility control. An injection scheme of water alternating gas (WAG) improves conformance (mobility control) in channeled settings.^2^ CO_2_-enhanced oil recovery (CO_2_-EOR) also enables long-term subsurface CO_2_ storage through dissolution, residual trapping, and, where applicable, structural or stratigraphic retention.^3^

From a theoretical perspective, CO_2_-EOR with associated CO_2_ storage can be framed as a coupled displacement and trapping process controlled by phase behavior, mobility, and reservoir architecture. An equation-of-state (EOS) description of the CO_2_–oil–brine system governs MMP and miscibility development, linking thermodynamic behavior to microscopic mechanisms such as swelling, viscosity reduction, extraction, and interfacial-tension reduction.^1^ At the field scale, the end point mobility ratio and relative permeability functions determine the stability of the displacement front and the need for mobility-control strategies such as WAG, tapered WAG, or hybrid schemes, particularly in channelized or stratified reservoirs.^2^ Storage performance is further influenced by how injected CO_2_ is partitioned between mobile, residually trapped, and dissolved phases, and by the fraction of injected mass ultimately retained in the subsurface rather than recycled to surface.^3^

This study focuses on a mature channel-belt sandstone reservoir in Colombia. A high-CO_2_ flare-gas stream, otherwise vented, is evaluated for subsurface injection using existing infrastructure. A compositional sector model was developed in tNavigator with a Peng–Robinson EOS calibrated to field PVT data. MMP at reservoir temperature was estimated using the Multiple-Mixing-Cell (MMC) method, and injection strategies were set to sustain miscible displacement. This work presents an evidence-based, simulation-ready framework for CO_2_-EOR with associated storage using on-site flare gas. The workflow integrates EOS calibration, MMP estimation, and the design and evaluation of CO_2_ injection schemes. Performance metrics include incremental oil production (Δ*N*p), recovery factor (*R*_F_), CO_2_ utilization (*U*_CO_2__), defined here as the mass of CO_2_ injected per incremental stock tank barrel of oil (kg CO_2_/stb-inc) and net CO_2_ stored. In addition, the study outlines requirements for well integrity and the repurposing of facilities for CO_2_ service, aligned with applicable standards such as those issued by NACE, API, and ISO.

## 2 Background

### 2.1 Theoretical Framework

CO_2_-EOR and its associated geological storage processes are governed by multiphase flow through porous media, coupled with compositional phase behavior. Fluid flow follows multiphase Darcy’s law, accounting for pressure and gravity effects. The flux of each phase is defined by eq 1.^4^

1 $$u_{\alpha }=-\frac{kk_{r\alpha }\left(S\right)}{\mu_{\alpha }}\left(▽p_{\alpha }-\rho_{\alpha }g\right)$$

where *u*_α_ is the superficial velocity of phase α∈{oil, water, gas}, *k* is the absolute permeability tensor, *k*_*r*α_(*S*) is the relative permeability to phase α as a function of phase saturations *S*, μ_α_ is the viscosity of phase α, *p*_α_ is the phase pressure, ρ_α_ is the phase density; and *g* is the gravitational acceleration vector. Saturation satisfies *Sw* + *So* + *Sg* = 1.^4^

Compositional simulators solve component mass balance equations, where mass transport is driven by the convective terms from eq 1 and coupled with thermodynamic phase behavior. The general component mass conservation is given by eq 2.^5^

2 $$\phi \frac{\partial \left(\rho x_{i}\right)}{\partial t}+▽.\left(\sum_{\alpha }\rho_{\alpha }x_{i\alpha }u_{\alpha }\right)=q_{i}$$

where ϕ is porosity, ρ is the mixture mass (or mole) density, *x*_*i*_ is the overall fraction of component *i*, *x*_*i*α_ is the composition of component *i* in phase α, *u*_α_ is given by eq 1, and *q*_*i*_ is the source/sink of component *i*.^5^

Phase equilibrium is enforced by equality of component fugacities obtained from EOS-based flash calculations. eq 3 expresses this equilibrium criterion^5^

3 $$f_{i}^{\left(o\right)}\left(T,p,x^{\left(o\right)}\right)=f_{i}^{\left(g\right)}\left(T,p,y^{\left(g\right)}\right)$$

where *f*_*i*_^(o)^ and *f*_*i*_^(g)^ are the fugacities of component *i* in oil and gas phases, respectively, *T* is temperature, *p* is pressure, and *x* ^(0)^,*y* ^(g)^are the phase-equilibrium computed via EOS-based flash calculations.^5^ This framework supports prediction of phase transport and interactions.

### 2.2 Miscibility and Displacement Efficiency

Above the MMP, dynamic miscibility driven by coupled vaporizing–condensing mass transfer enhances displacement efficiency by reducing residual oil saturation. Below MMP, CO_2_ still swells and thins oil, but recovery remains limited by capillary forces.^6^

Empirical correlations (e.g., Yellig–Metcalfe, Alston, Glaso, Cronquist) provide rapid MMP estimates but becomes unreliable outside their calibration ranges, particularly for impure CO_2_ streams.^6^ This study applies the MMC method with a tuned EOS to compute MMP. Miscibility is reached when the equilibrium tie-line between phases collapses to zero length in composition space, as expressed in eq 4.^7,8^

4 $$L_{p,n}=∥x^{\left(o,n\right)}-y^{\left(g,n\right)}|{|}_{2}\underset{n\to \infty }{\to }0\Rightarrow p\geq MMP$$

where *L*_*p*,*n*_ is the dimensionless tie-line length at iteration *n*, *x* ^(,*n*)^,*y* ^(*n*)^ are the liquid and vapor phase compositions from EOS flash calculations at pressure *p*.^7,8^

A critical design parameter is the end point mobility ratio, which evaluates the risk of viscous fingering during injection. It is defined in eq 5.^2^

5 $$M=\frac{\left(k_{rg}/\mu_{g}\right)}{\left(k_{ro}/\mu_{o}\right)},$$

where *M* is the end point mobility ratio, *k*_rg_ and *k*_ro_ are the end point relative permeabilities of gas and oil, evaluated at residual oil saturation and irreducible water saturation, respectively, and μ_*g*_, μ_*o*_ are their viscosities. Values *M* ≈ 1 are desirable; *M* ≫ 1 signals viscous fingering and gravity override and motivates mobility control strategies such as WAG or foam injection.^2^

### 2.3 In Colombia

In Colombia, cluster-based screening has identified five CCS-EOR opportunities across four subsurface clusters, with an estimated storage capacity of 142 Mt CO_2_ and 465 MMbbl of incremental oil, potentially mitigating approximately 25% of the oil sector’s projected 2025–2040 CO_2_ emissions.^9^ The national oil company, Ecopetrol, has integrated CO_2_ injection into its decarbonization strategy, advancing feasibility studies and pilot designs within a national subsurface-cluster framework for CO_2_-EOR and dedicated storage. Public targets include launching the first CO_2_-injection pilot by 2025 and pursuing 344 MMbbl of incremental oil and 280 Mt CO_2_ storage capacity across prioritized assets, subject to feasibility and approvals.^10^ To date, development remains at the evaluation and pilot-planning stages, progressing in regulatory aspects and environmental permits.^9^

### 2.4 Case Studies

Representative field projects were reviewed to benchmark CO_2_-EOR and storage performance. In the Wasson–Denver Unit (Permian, USA), a long-term miscible WAG flood using 96.9% CO_2_ achieved 60.5% net retention to 2013 ^11^ and delivered an incremental oil recovery of 11% for the Denver Unit.^12^ Other Permian analogues report incremental recovery factors of 8–25% and CO_2_ retention of 55–65% with gas recycling.^12^ In Dulang (Malaysia), a recycle stream containing 50% CO_2_ under immiscible WAG delivered an incremental recovery of 5–7% in the pilot area, demonstrating performance uplift below MMP.^13^ In Jilin Oilfield (China), miscible CO_2_ WAG using high-purity gas attained 8–15% incremental oil recovery per block and about 80% retention.^14^ In the Middle East, ADNOC implemented a miscible WAG achieving 12% incremental oil recovery.^15^ In Colombia (Llanito/Galán), small-scale cyclic (huff-and-puff) pilots in Galán (1989–1992) and Llanito (2008) demonstrated incremental oil recovery at laboratory and well scale, though public data on full-field performance is limited.^16^

## 3 Geological and Reservoir Description

The study targets a meandering fluvial-channel sandstone in a mature Colombian onshore field under waterflood since 2014, with three producer and three injector wells (Figure 1). Channel geometry is constrained by 3D seismic data and reveals a laterally continuous sand body capped by regionally thick shales that provide an effective top seal. These geological constraints informed the compositional sector model built in tNavigator [21].

**Figure 1:**
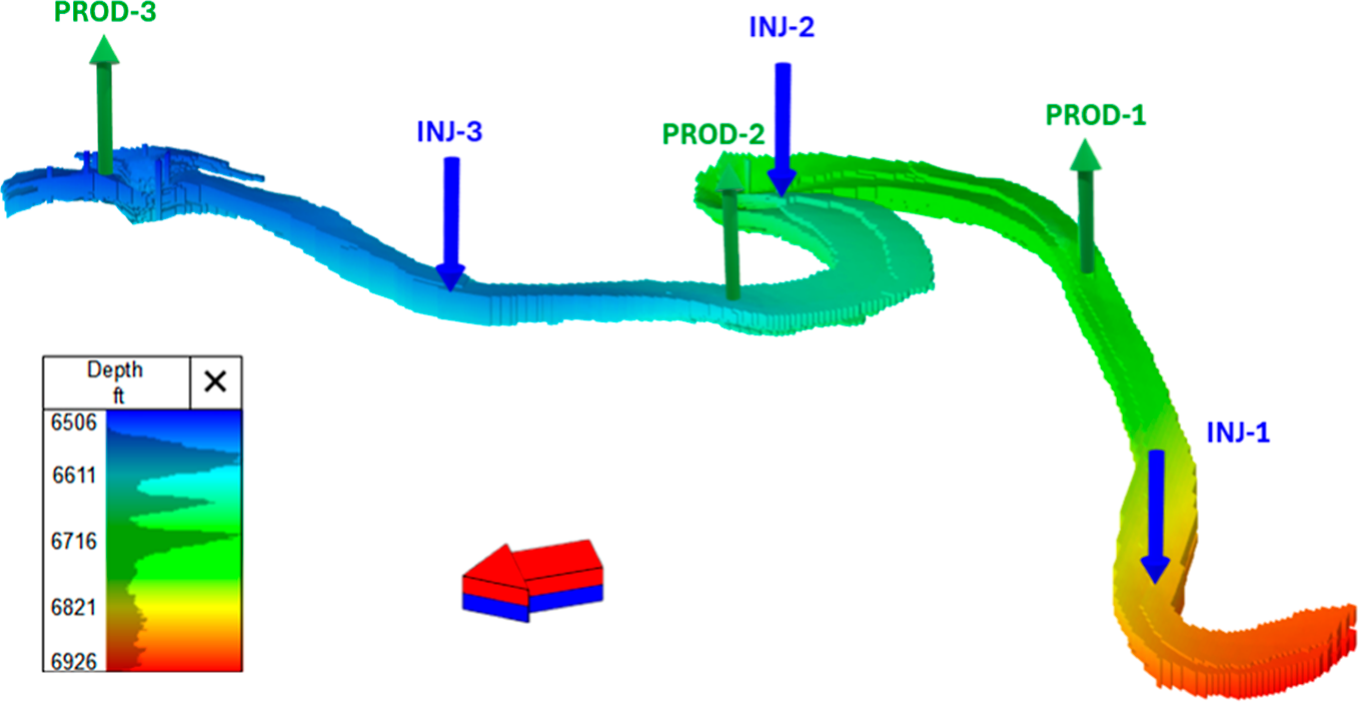
3D sector model of the reservoir showing producer–injector layout and depths (TVDSS); constructed using tNavigator software.

Cumulative field performance to 2025 indicates a recovery factor of 20.4%, including 13.4% primary and 6.9% secondary recovery [21]. The limited areal sweep and screening outcomes motivate the evaluation of CO_2_-EOR to enhance the microscopic displacement and macroscopic conformance.

The reservoir consists of clean to moderately shaly channel sands with local shale drapes acting as baffles that generate heterogeneity between flow units but maintain favorable storage and flow capacity. The permeability anisotropy indicates restricted vertical communication consistent with a channeled architecture. Fluids are a medium-grade, initially undersaturated oil with a low gas content and low-salinity formation water. Reservoir and fluid parameters are summarized in Table 1.

**Table 1:** Reservoir and Fluid Properties Reproduced with Permission From SierraCol Energy^17^

category	parameter	value/range
depth	true vertical depth subsea (TVDSS)	average 6690 ft (6926–6506 ft)
	water–oil contact (TVDSS)	6865 ft
reservoir quality	porosity (ϕ)	average 19.8%
	permeability (*K*)	950 mD (101–4565 mD)
	permeability ratio (*K*_*v*_/*K*_*h*_)	0.34
	net pay	28 ft (single layer)
	rock type	sandstone with kaolinite and microcrystalline quartz
reservoir conditions and fluids	initial pressure	3200 psi
	current pressure	3500 psi
	temperature	205 °F
	oil gravity	29.6 °API
	oil viscosity (μ_*o*_)	3–5 cP
	bubble point pressure (*P*_b_)	67.7 psi
	solution GOR (*R*_s_)	9.4 scf/stb
	oil formation volume factor (*B*_o_)	1.05 RB/stb
	formation-water salinity	200–600 ppm

## 4 Fluid System and EoS Development

### 4.1 Fluids Characterization and EoS Tuning

Two fluid samples under reservoir conditions were used to characterize live oil and set calibration targets in tNavigator software. A downhole sample contained N_2_, CO_2_, C_1_–C_6_, and a C_7_^+^ fraction (93.9 mol %). Heavy ends were represented by partitioning C_7_–C_20_^+^ pseudocomponents from a surface sample, scaled to match the downhole C_7_^+^ total. The injectant corresponds to the facility flare-gas stream (50.74 mol % CO_2_). A 25-component Peng–Robinson EoS was calibrated to reproduce *P*_b_, *R*_s_, *B*_o_, μ_o_, and ρ_o_. Figure 2 shows that EoS predictions closely match laboratory data, validating the model for MMP and compositional simulations.

**Figure 2:**
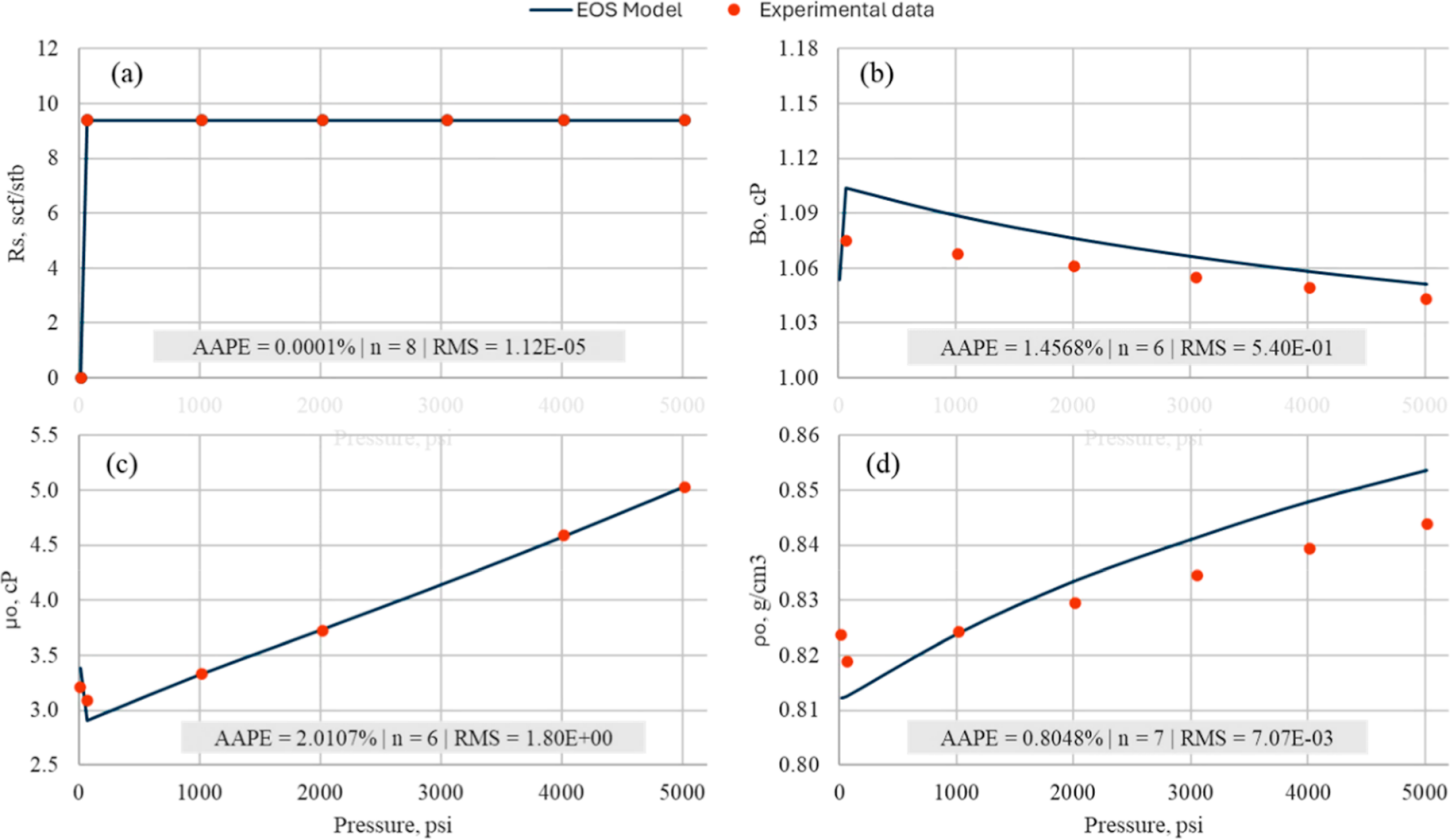
Reservoir fluid properties (a) *R*_s_, (b) *B*_o_, (c) μ_o_, (d) predicted using PR-EoS in tNavigator software (blue), history matched with the laboratory PVT data (orange) at 205 °F ρ_o_.

### 4.2 Minimum Miscibility Pressure (MMP) with Flare Gas

The MMP was estimated in PVT Designer (tNavigator) using the MMC method, which equilibrates injected gas with reservoir oil across pressure steps and diagnoses miscibility from the collapse of the equilibrium tie line length. As the MMC method captures repeated mass transfer between oil and gas, the estimated value corresponds to a multicontact MMP rather than a first-contact miscibility pressure. This approach is widely used as an analytical proxy for slim-tube measurements and correlates well when dispersion is considered.^7^ Using the tuned EoS fluids and the CO_2_-rich flare gas (50.74 mol %) as injectant, the estimated multicontact MMP is 2600 psi at 205 °F. This value is below the field operating envelope and therefore supports a miscible CO_2_ process with the available gas. Although MMP generally increases with temperature, the limited vertical extent of the sector (about 6506–6926 ft TVDSS) suggests that any depth-related variation would be modest and would not change the conclusion that miscible operation is feasible within the field pressure envelope. Figure 3 shows CO_2_ solubility in oil and tie line length versus pressure. The MMP is identified at the pressure where the tie line length approaches zero.^8^

**Figure 3:**
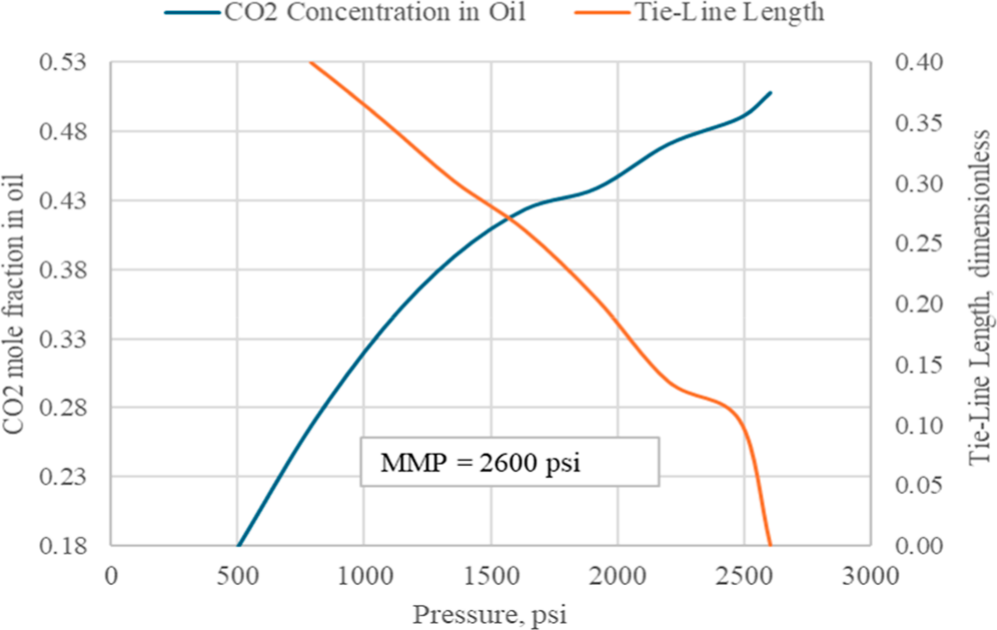
Minimum miscibility pressure (MMP) predicted through MMC analysis using tNavigator - CO_2_ mole fraction in oil (left) and tie-line length (right) versus pressure.

## 5 Simulation Design and Scenarios

### 5.1 Sector Model

A 3D compositional sector model was developed in tNavigator to support all CO_2_-injection forecasts. The model comprises 117 × 293 × 25 grid cells (857,025 total) over an extent of approximately 4709 ft × 8855 ft × 424 ft, with a mean cell size of 33.2 × 33.6 × 2.86 ft (I × J × K). The grid uses corner-point geometry. Forecast results were reported at monthly intervals, while the simulator used automatic adaptive time stepping, with time-step reduction and restarts triggered when required to maintain Newton convergence. Internal timesteps varied from as low as about 0.001 days during difficult convergence periods to values of the order of days in smoother intervals. The CO_2_-injection scenarios were computationally intensive, with typical runtimes of about 30 to 50 h per scenario, reflecting the cost of the EoS-based compositional formulation, the 25-component fluid description, repeated phase-equilibrium calculations, and convergence control over a long forecast period.

Material-balance initialization yields *OOIP*5.109 MMstb, *OWIP* 2.872 MMstb, and *OGIP* 13.18 MMscf. Total pore volume is 8.78 MRB, with 64.7% hydrocarbon-filled and 28.4% displaceable. Relative-permeability and capillary-pressure functions represent the waterflooded state with end points *S*_wc_ = 33% and *S*_orw_ = 40.0%. Oil–water and gas–oil relative permeability curves were incorporated as shown in Figure 4, capturing the expected gradual reduction in oil relative permeability with increasing water and gas saturations, and the corresponding increase in water and gas mobilities. These functions ensure a realistic representation of displacement efficiency and phase mobility behavior under both waterflooding and gas injection conditions.

**Figure 4:**
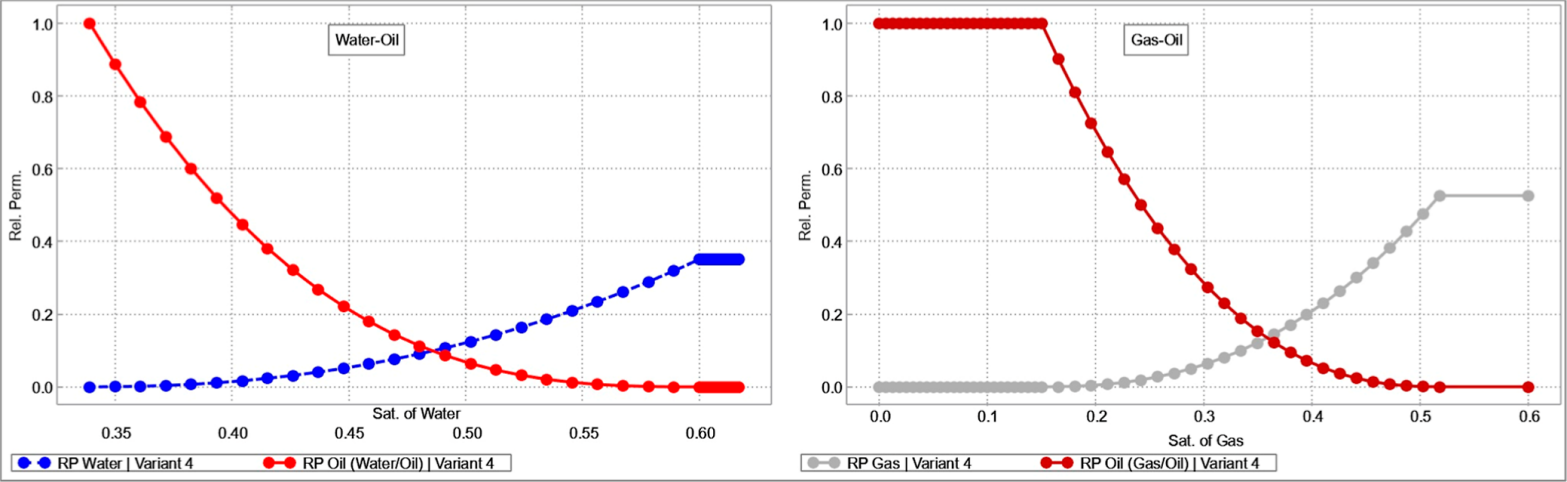
Oil–water and gas–oil relative permeability curves used in the simulation model, constructed using tNavigator.

History matching covered January 2007–March 2025, using field totals and per-well rates. The model accurately reproduced production, injection, and pressure under waterflood conditions. Figure 5 presents field rate matching and Figure 6 cumulative volumes. Field-scale deviations remain below 0.3% for liquids and 16.8% for oil, which is acceptable given long-term metering uncertainties. This calibration establishes a robust dynamic foundation for subsequent CO_2_-EOR forecasting.

**Figure 5:**
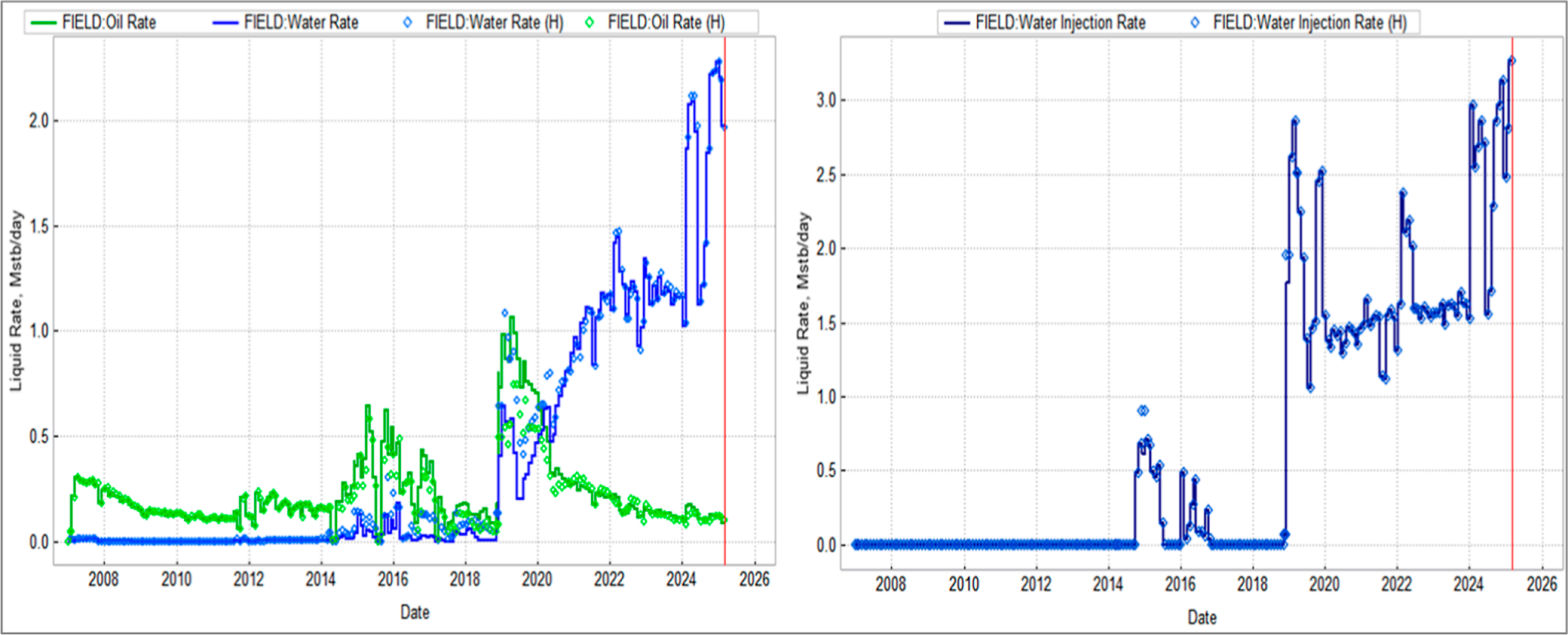
Field history match showing simulated (lines) and observed (markers); left: field oil and water production; right: water-injection rate.

**Figure 6:**
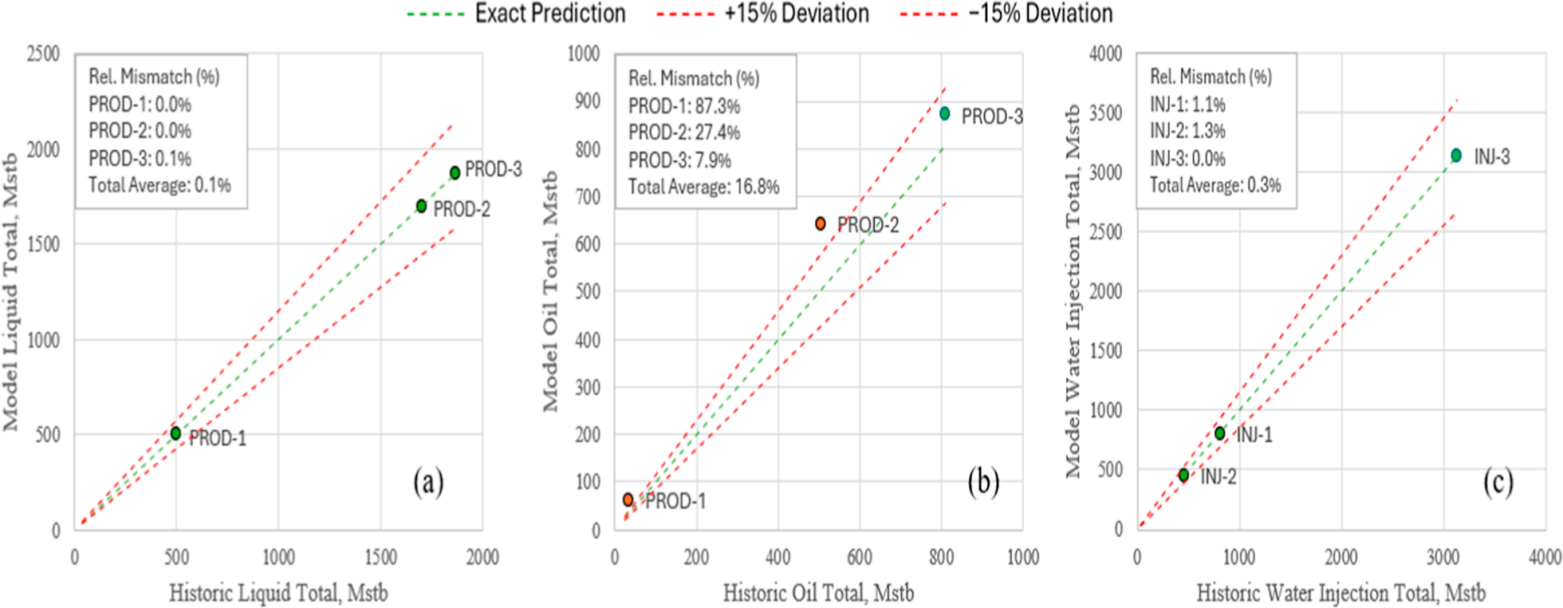
Comparison of simulated and historical cumulative volumes by well: (a) total liquids, (b) oil, (c) water injection. Green line shows 1:1 reference; dashed red lines indicate ±15% bounds.

### 5.2 Well Controls and Operating Envelope

Production wells were operated under bottom-hole pressure (*BHP*) control, while injectors were limited by a fracture-safe ceiling derived from the step-rate test (SRT) conducted at INJ-3. Vertical flow performance tables for gas injectors were generated using tNavigator to ensure stable operation under both *BHP* and rate control. Facility handling limits for CO_2_-rich gas and water were applied at the group level. Set points and constraints are summarized in Table 2. The injection envelope follows the 0.9*xP*_frac_ safety criterion, using the fracture gradient interpreted from the SRT shown in Figure 7 and adjusted to each well’s perforation depth.

**Table 2:** Well Controls and Operating Envelope^a^ ^b^

well/group	role	control	limit	allocation/rule
PROD-1	producer	*BHP*_*min*_	3502 psi	economic limit 2 stb/d
PROD-2			3491 psi	
PROD-3			3371 psi	
INJ-1	injector	*BHP*_*max*_	4292 psi	water-dominant; below contact
INJ-2			4177 psi	priority gas injectors
INJ-3			4132 psi	
CO_2_-rich gas rate	group	cap	800 Mscf/d	preferentially to INJ-2 and INJ-3
water injection		cap	3343 BWPD	split: INJ-3 1700; INJ-1 1400; INJ-2243

a PROD-1 to PROD-3 are production wells, and INJ-1 to INJ-3 are injection wells.

b Cap: group controls for CO2-rich gas injection and water rates are capped to the stated limits.

**Figure 7:**
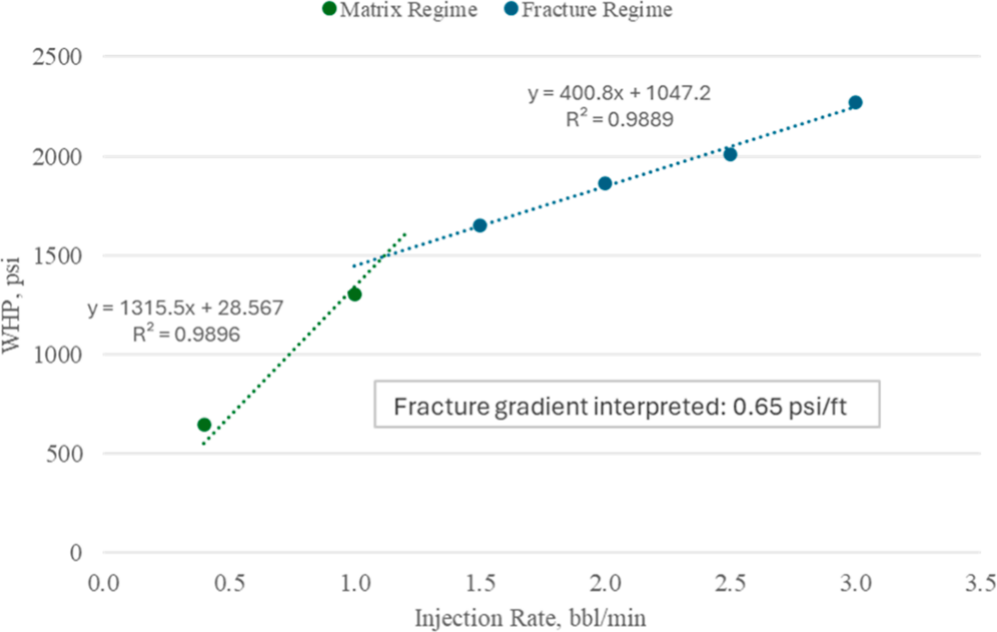
SRT at INJ-3 showing WHP (Well Head Pressure) versus rate. The interpreted fracture gradient is 0.65 psi/ft.

### 5.3 Scenario Schedule and Forecast Strategies

All cases respect the operating envelope in Table 2; differences arise only from injection timing and CO_2_ allocation, ensuring that performance reflects strategy rather than constraints. From March to September 2025, the field remains on waterflood, CO_2_ injection starts in October 2025 for scenarios S1–S6, while S0 serves as the waterflood reference. The evaluation focuses on cumulative oil to 2036, CO_2_ utilization per incremental barrel, WOR and GOR stability, produced-to-reinjected CO_2_ balance, and net CO_2_ stored. Table 3 summarizes schedules and settings for each forecast scenario.

**Table 3:** Scenario (S0 to S6) Schedules and Injection Settings

ID	window (months)	strategy	CO_2_ allocation [INJ-1/INJ-2/INJ-3], Mscf/d	WAG (W/G, cycle d)
S0 (Base)	134	waterflooding	0/0/0	n/a
S1	127	CGI INJ-2&3 (INJ-1 water)	0/250/550	n/a
S2	127	WAG INJ-2&3 (INJ-1 water)	0/434/366	1:1, 60/60
S3	F1:8	WAG All INJ(INJ-1 delayed)	F1: 0/500/300	1:1, 60/60
	F2:119	INJ-1 gas starts in F2	F2: 50/450/300	
S4	F1:20	tapered WAG All INJ (1:1→ 3:1)	F1: 0/500/300	F1: 1:1, 60/60
	F2:36	tapered WAG (water fraction increases F1 → F3)	F2: 50/450/300	F2: 2:1, 60/30
	F3:71		F3: 100/400/300	F3: 3:1, 90/30
S5	slug: 10	CGI → WAG All INJ	slug: 0/434/366	slug: CGI; then 1:1, 60/60
	WAG: 117		WAG: 50/450/300	
S6	slug: 10	Role-Swap CGI → WAG	slug: 400/0/400	slug: CGI; then 1:1, 60/60
	WAG: 117	PROD-1 → INJ-1′	WAG: 400/0/400 (INJ-1 shut-in)	
		INJ-2 → PROD-1′		

Each scenario was designed to identify the most effective CO_2_-injection strategy under the defined operating envelope. The rationale and intended purpose of each case are summarized as follows, highlighting how variations in scheduling and well configuration aim to optimize recovery and storage performance.

- S0—waterflood continuation: baseline for ongoing operations and depletion trend.
- S1—CGI: continuous CO_2_ injection at INJ-2/3 while maintaining INJ-1 on water for pressure support.
- S2—WAG at INJ-2/3: concentrate CO_2_ where remaining oil is highest while keeping a water curtain at INJ-1 to manage mobility and GOR.
- S3—WAG INJ-1/2/3: stabilize sweep via INJ-2/3, add INJ-1 gas later to access upper zone with controlled GOR.
- S4—tapered WAG: increase water fraction over time to reduce late-time recycling while sustaining pressure support.
- S5—Hybrid CGI → WAG: establish miscible contact via 10-month CO_2_ slug (0.13 PV), then WAG for mobility/conformance control.
- S6—Role-swap CGI → WAG: exchange well roles to reroute sweep and test impacts on breakthrough timing and sweep efficiency.

## 6 Simulation Results and Discussion

### 6.1 Recovery and Displacement Efficiency

Forecasts for all scenarios were run under Table 2 controls. Figure 8 shows the field cumulative production. *N*p and recovery factor, *RF* at 2036, every CO_2_ case outperforms the waterflood base (S0 with greatest gains from hybrids initiating a 10-month CO_2_ slug followed by WAG).

**Figure 8:**
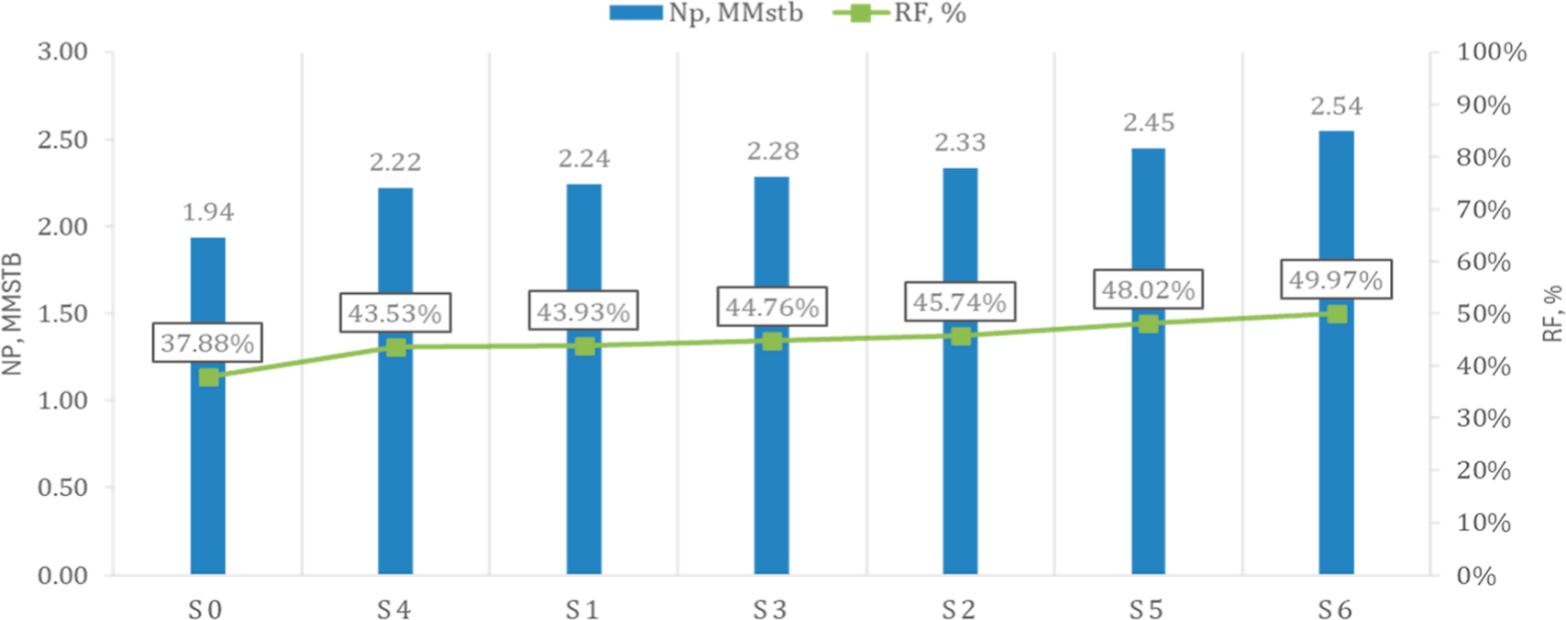
Field cumulative oil (Np) and recovery factor (*R*_F_) in 2036. Bars show *N*p; the overlaid green line shows *R*_F_.

The strongest result is S6, in which a 10-month continuous-gas slug precedes WAG at INJ-1′ and INJ-3. A role-swap between wells redirects the front from PROD-1 to INJ-1′ and from INJ-2 to PROD-1′, aligning injection with corridors of higher remaining oil saturation. During the slug, INJ-1 provides a water curtain to maintain pressure support, then is shut in to limit early recycling. The shut-in of PROD-1′ was implemented after sustained gas-breakthrough behavior became evident during the forecast (no single fixed GOR threshold was imposed; the decision was associated with sustained high gas–oil ratios consistent with gas-breakthrough conditions), with the well remaining shut in through the end of the simulation to reduce early recycling and improve areal sweep toward PROD-2. From an implementation perspective, such a role-swap configuration would require dedicated assessment of wellbore condition, completion modifications, and associated intervention time and cost before field deployment. These practical conversion aspects are outside the scope of the present study and are being examined in separate work. Configuration changes and flow evolution are illustrated in Figures 9 and 10.

**Figure 9:**
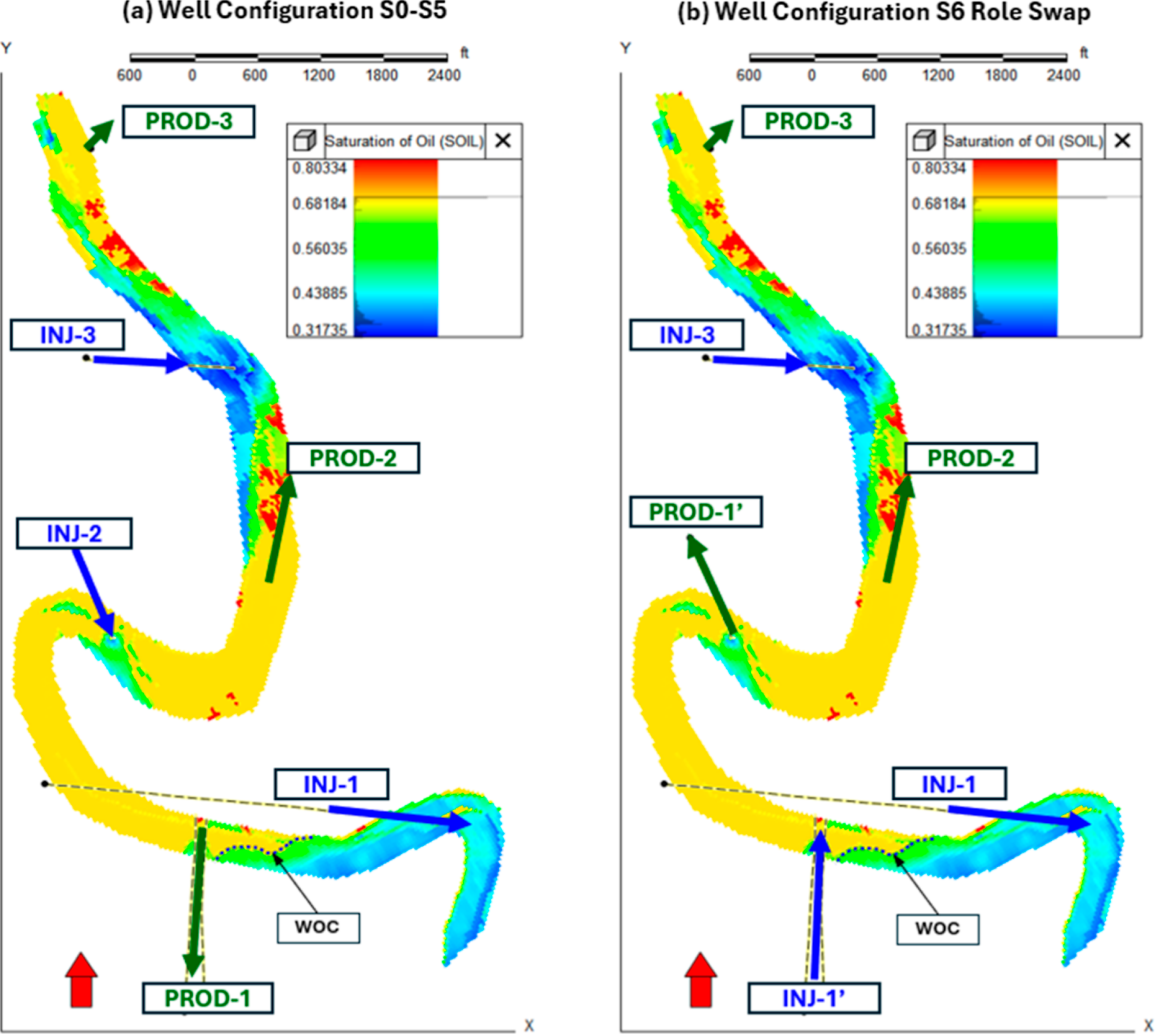
Initial oil saturation and well configuration at gas-injection start. (a) Waterflood configuration (S0–S5). (b) Role-swap in S6. Black dashed line marks the *WOC* at 6865 ft TVDSS.

**Figure 10:**
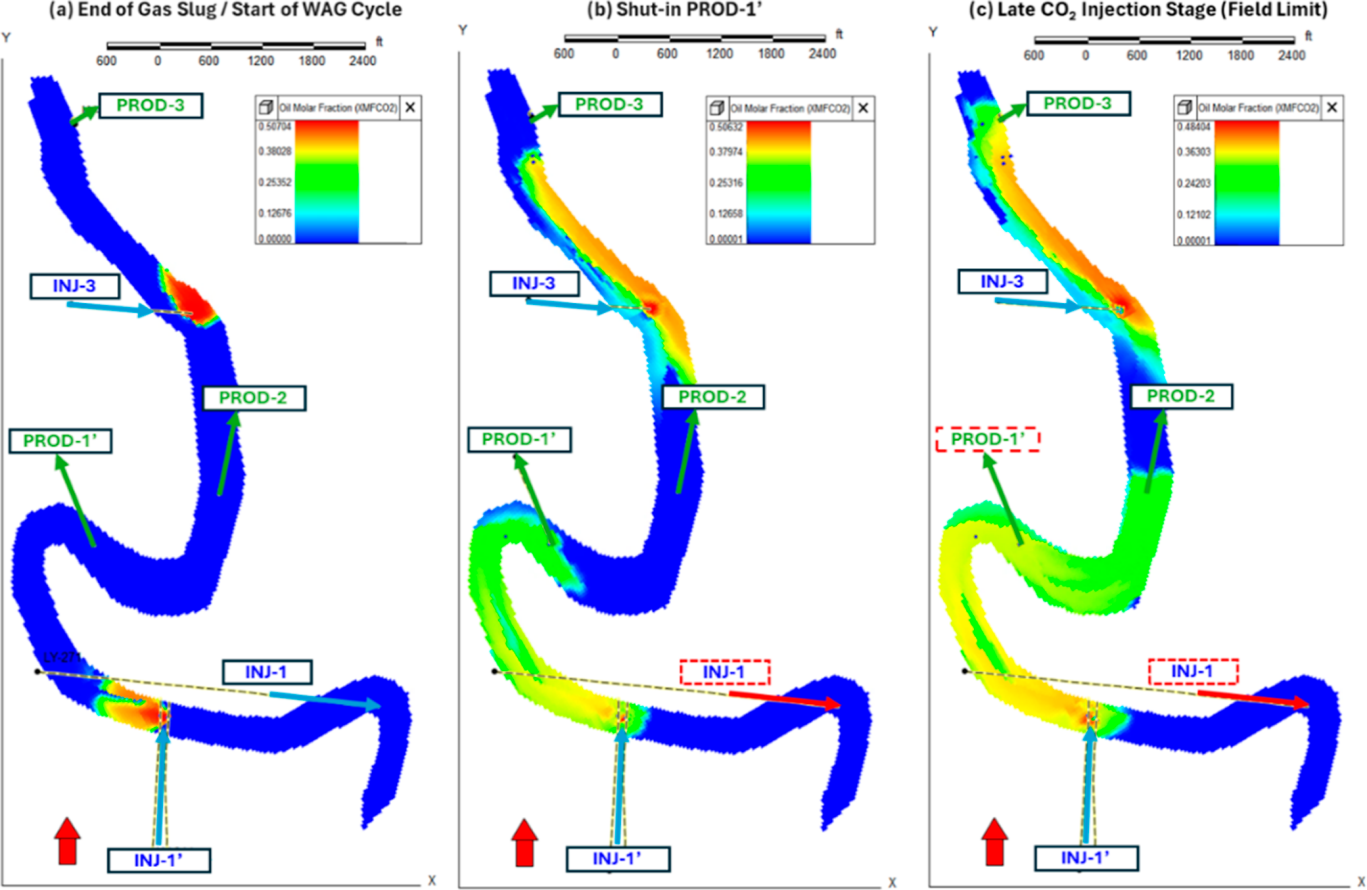
CO_2_ distribution evolution. (a) Transition from slug to WAG at INJ-1′ and INJ-3. (b) Redistribution after shutting PROD-1′, promoting lateral sweep toward PROD-2. (c) Late-time stage showing expanded contact.

As a complementary diagnostic, this study uses miscibility contacted pore volume (*MCPV*) to indicate the extent of pore volume newly contacted by injected CO_2_ under miscible conditions. To gauge contacted volume, *MCPV* was calculated by counting grid cells where the oil-phase CO_2_ mole fraction, $x_{{Co}_{2}}^{oil}$ , exceeds its preinjection maximum threshold (0.00235 CO_2_ mole fraction). This threshold was used as a background cutoff to distinguish newly CO_2_-contacted cells from the initial in situ CO_2_ content. Figure 11 reports recovery outcomes in 2036, where Δ*N*p denotes incremental cumulative oil relative to the continued-waterflood base case (S0). A higher contacted pore volume usually corresponds to greater oil gain, though not always proportionally, contact in low-quality rock or near early water breakthrough may enlarge the metric without equivalent production response, reflecting heterogeneity or channeled gas flow. *MCPV* therefore, complements CO_2_-utilization and recycling metrics discussed in Section 6.2.

**Figure 11:**
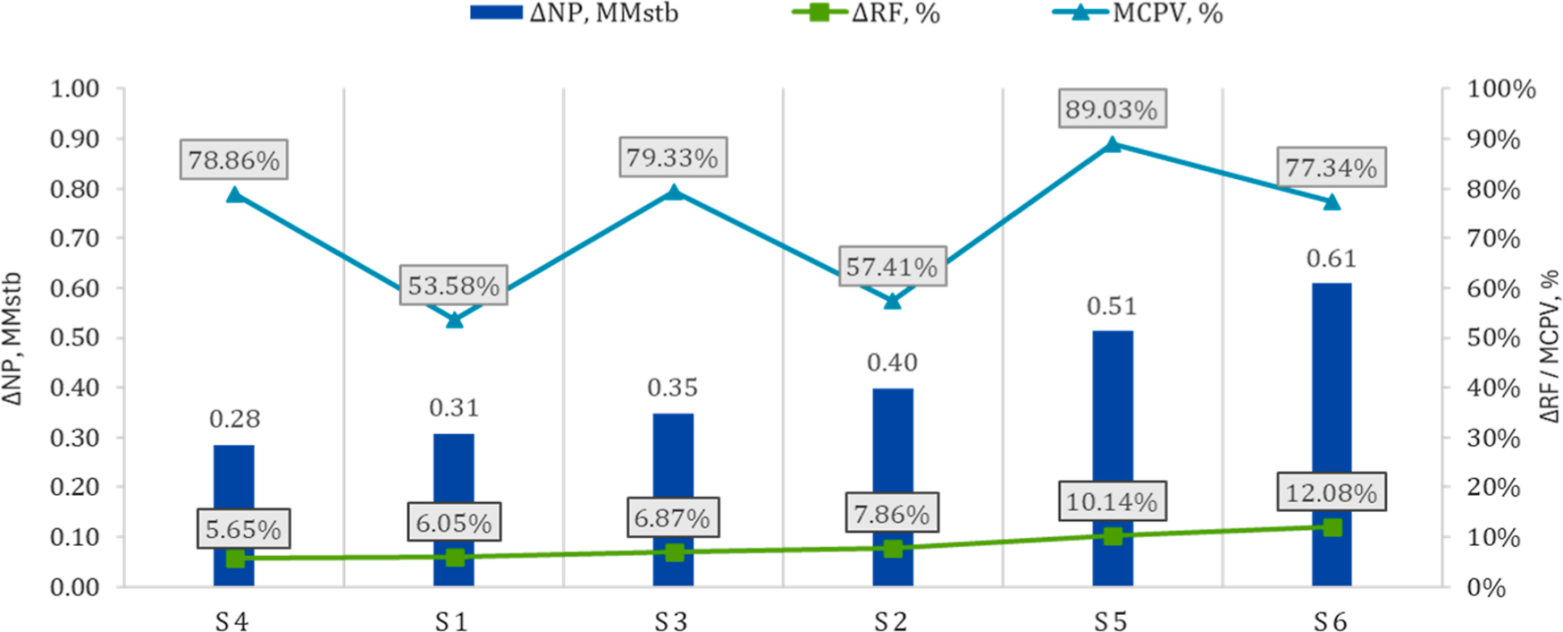
Incremental performance at 2036 relative to S0. Bars show Δ*N*p; the green line shows Δ*R*_F_; the blue line shows MCPV.

Overall, S6 achieves the highest *N*_p_ and *R*_F_ by 2036, as illustrated in Figure 8, and exhibits the largest incremental response as shown in Figure 11. Indicating that front redirection, a finite slug to establish miscibility, a temporary water curtain, and selective producer shut-ins act synergistically to enhance macroscopic sweep and recovery.

### 6.2 CO_2_ Storage and Utilization Efficiency

All CO_2_ quantities discussed in this section were obtained directly from the simulator as component mass (kt CO_2_), so no conversion from surface or reservoir volume reference conditions was required. Net CO_2_ stored is calculated as the difference between the total CO_2_ mass in place at the end of the forecast (2036) and the initial CO_2_ inventory, thereby excluding CO_2_ originally present in the reservoir. The CO_2_ utilization efficiency (*U*_CO_2__) is defined in eq 6 as the total injected CO_2_ mass, *M*_CO_2__, which includes recycled volumes, divided by the incremental oil produced relative to the waterflood base Δ*N*p. Lower *U*_CO_2__ values denote higher efficiency, as less CO_2_ is required per incremental barrel.

6 $$U_{{CO}_{2}}=\frac{M_{{CO}_{2}}}{\Delta Np}$$

CO_2_ retention (*R*_CO_2__) is defined here as the ratio of net CO_2_ stored to total CO_2_ injected; recycled CO_2_ is not excluded from the denominator. For the CO_2_ balance shown in Figure 12, recycled CO_2_ was estimated as the difference between the total CO_2_ injected and net CO_2_ stored.

**Figure 12:**
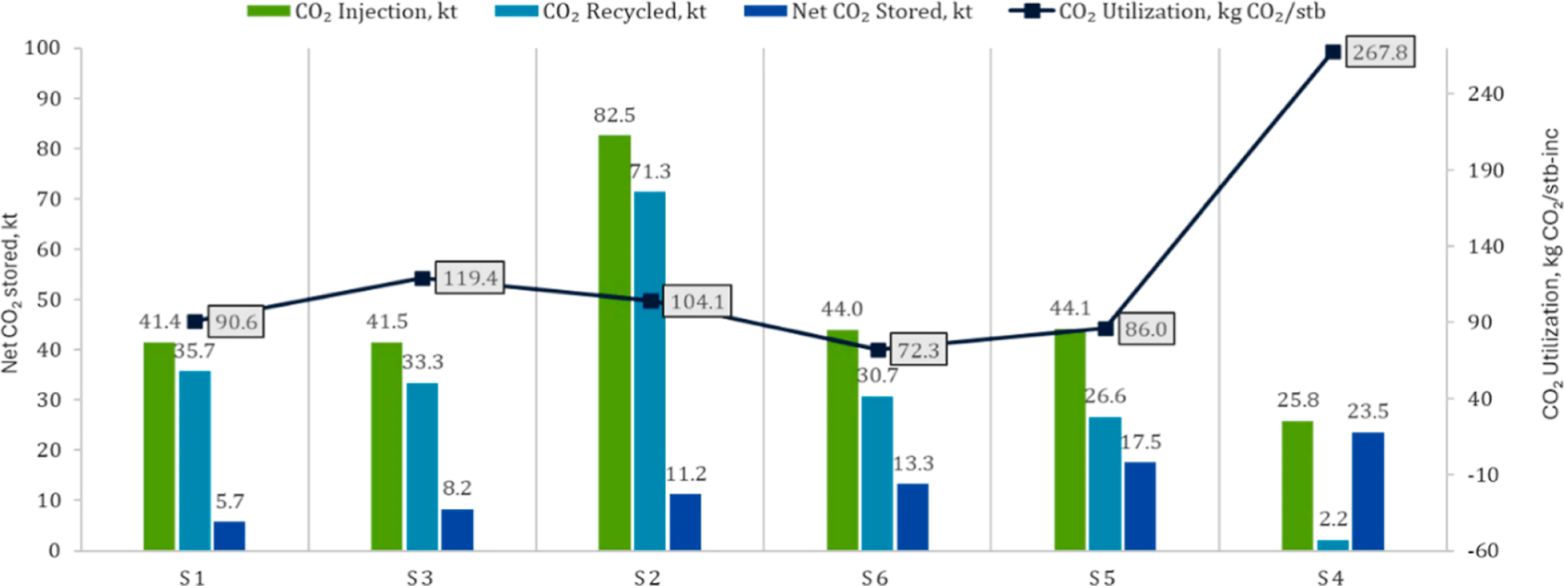
CO_2_ balance and utilization. Green bars: injected; teal: recycled; dark blue: net stored. Black line: utilization (*U*_co_2__).

Figure 12 compares the CO_2_ mass balance across scenarios. The tapered WAG (S4) stores the most CO_2_ (23.5 kt) but shows the poorest utilization performance (268 kg CO_2_/stb-inc). Its water-dominant cycles curb late recycling but limit gas throughput and incremental oil, with gas entry below the WOC at INJ-1, thereby enhancing storage over recovery. In contrast, S6 (Role-swap CGI→WAG) achieves the best utilization (72 kg CO_2_/stb-inc) with moderate CO_2_ storage (13.3 kt), consistent with its superior recovery. The role swap, finite slug, and selective producer shut-in reduce early recycling and direct CO_2_ through high-value oil zones, converting a greater gas fraction into incremental barrels.

Intermediate > cases (S1–S3) exhibit higher gross injection but limited storage improvement, for example, S2 injects 82.5 kt and recycles 71.3 kt CO_2_, inflating *U*_CO_2__through repeated circulation. The nonmonotonic relation between utilization and storage indicates a clear trade-off: recovery-oriented schemes such as S6 achieve low utilization but retain less mass, whereas storage-oriented designs such as S4 sequester more CO_2_ at the expense of efficiency. Net CO_2_ retention ranges from 13.6% to 91.3%, with S4 achieving the highest retention (91.3%) and S1 the lowest (13.6%).

For development planning, CO_2_ storage and barrel efficiency targets should be optimized jointly. Strategies focusing solely on storage may underperform economically, whereas recovery-driven designs may miss the sequestration objectives. Integrated evaluation ensures a balanced carbon and production performance.

### 6.3 Reservoir Pressure and GOR/Watercut Behavior

Figures 13 and 14 summarize the field response before and after CO_2_ injection began. Once injection begins, cumulative oil production diverges from the waterflood base case, with S6 exhibiting the steepest increase and highest recovery, a behavior also reflected in the *R*_F_ trend. Gas–oil ratio rises at late time for all strategies as gas mobility increases near producers, peaking in the CGI (S1). The tapered WAG (S4) mitigates this rise through progressively larger water fractions. Watercut stabilizes after gas injection, halting the upward trend observed under waterflooding. Oscillations in *GOR* and Watercut reflect alternating gas–water cycles: gas slugs temporarily enhance gas mobility, while water slugs restore liquid flow.

**Figure 13:**
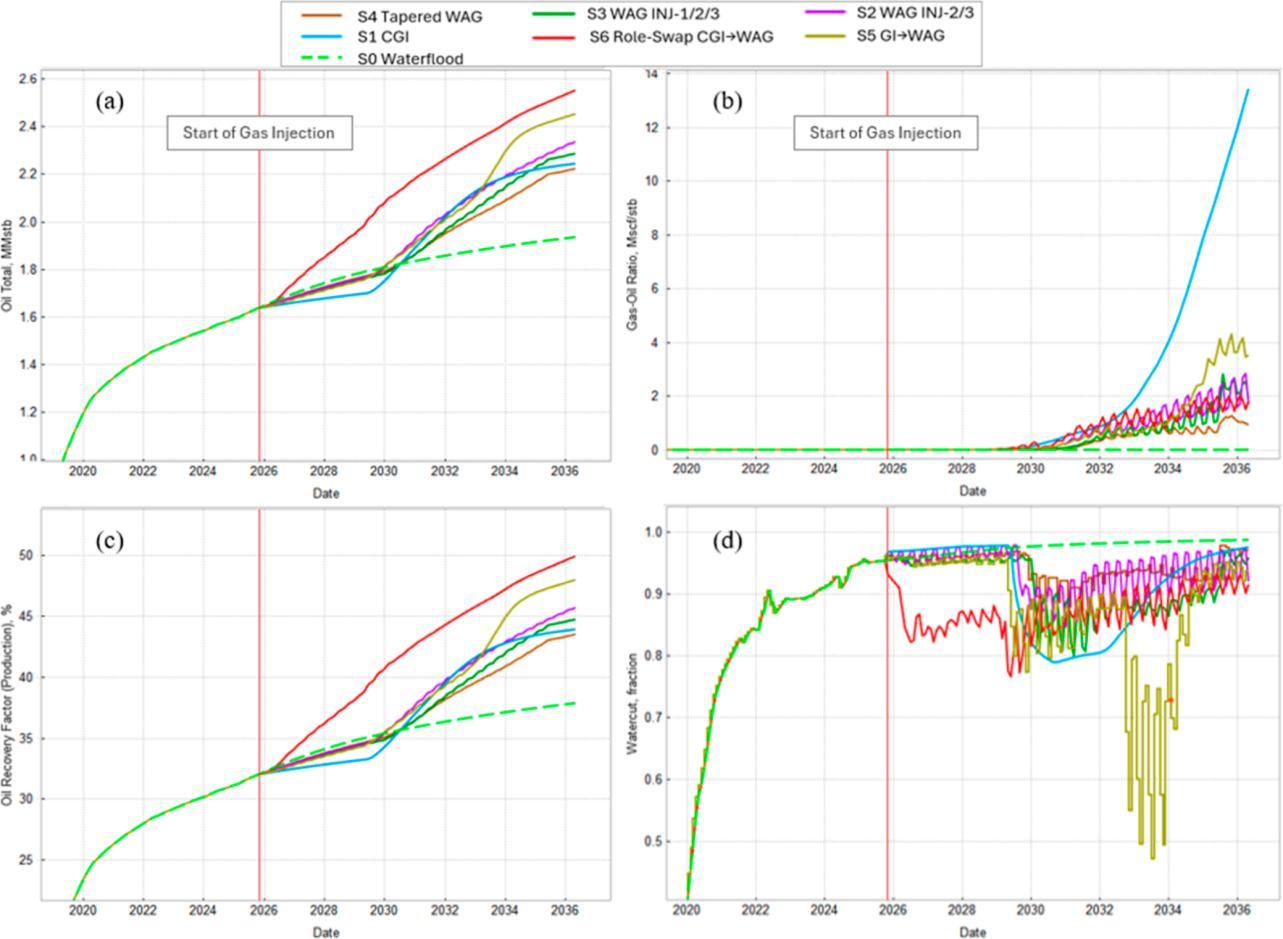
Field time series. Red vertical line: start of gas injection. (a) *N*p, (b) *GOR*, (c) *R*_F_. (d) Watercut. Waterflood base (green dashed) is compared with all gas-injection scenarios.

**Figure 14:**
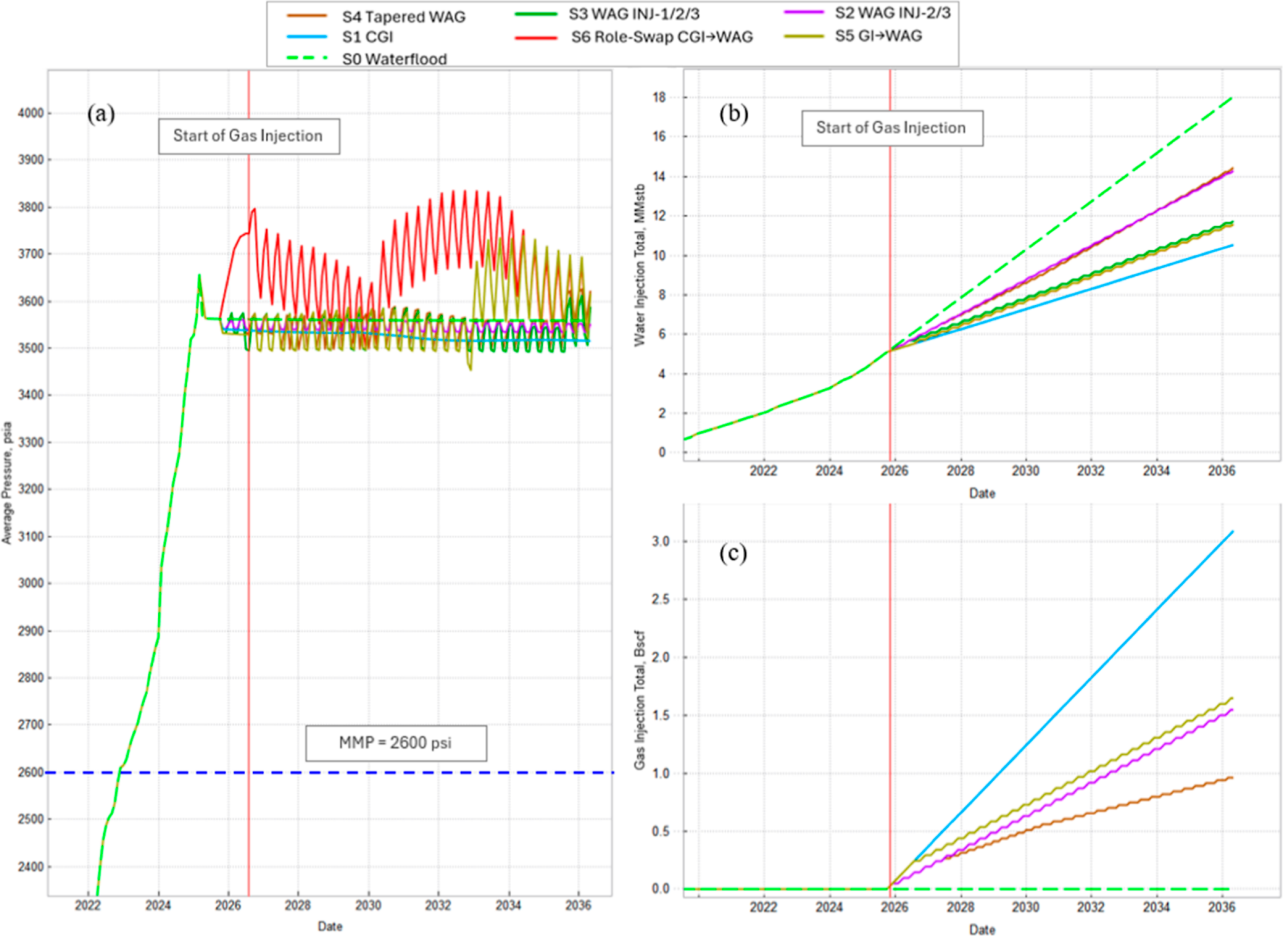
Field pressure and injection totals. (a) Average pressure. Red line: gas start; blue dashed line: MMP. (b) Cumulative water injection. (c) Cumulative gas injection.

Figure 14 confirms that all of the cases remain within the operating envelope. The average reservoir pressure stays above the MMP and below the fracture range, demonstrating miscible displacement without fracture risk. Cumulative water and gas injections show that CO_2_ cases require less water and gradually build gas throughput. Overall, the results demonstrate the trade-off between maximizing incremental oil (linked to higher late-time GOR) and controlling gas production via tapered or delayed WAG cycles.

## 7 Conclusions and Recommendations

Compositional forecasts demonstrate that operating above the MMP enables stable miscible displacement in all CO_2_-injection scenarios. Clear improvements are observed in cumulative oil and recovery factor, with incremental gains between 5.6% and 12.0% over continued waterflooding. The strongest uplift occurs in hybrid schemes that initiate contact with a CO_2_ slug followed by WAG. The role-swap CGI → WAG configuration provides the best recovery performance: front redirection, a short water-curtain phase, and selective producer shut-ins reduce early recycling and direct the gas through higher-value zones, resulting in the highest utilization (72 kg CO_2_/stb-inc) and moderate storage (13.3 kt). The tapered WAG maximizes storage (23.5 kt) but with weaker utilization performance (268 kg CO_2_/stb-inc) and smaller oil gain, consistent with water-dominant cycling and partial gas injection below the contact. Net CO_2_ retention ranges from 13.6% (S1) to 91.3% (S4), with S6 combining best utilization and 30.1% retention. Based on these results, development planning should jointly optimize CO_2_ storage and barrel efficiency. Storage-focused schemes may underperform economically, while recovery-driven ones may fall short of sequestration targets. Integrated evaluation is essential to balance carbon and production goals.

The outcomes of this study are consistent with those of international CO_2_-EOR experiences. Reported field applications across the Permian Basin, Jilin, ADNOC Onshore, and other miscible WAG projects demonstrate comparable trends in incremental recovery and CO_2_ retention. Colombian pilots, though still precommercial, further confirm technical feasibility and short-term injectivity using available CO_2_ sources. Overall, the evaluated CO2 injection strategies align with global benchmarks, validating the reliability of the proposed design framework and its applicability to the local reservoir conditions.

Repurposing waterflood facilities for CO_2_-EOR requires compliance with CO_2_ service standards and strong integrity management. The injected stream must remain dry to prevent corrosion, with inhibitors applied if moisture is present. Wells should meet NACE MR0175/ISO 15156 using corrosion-resistant materials and a CO_2_-stable cement. Surface equipment must include CO_2_-rated components per API 6A, and system integrity must be monitored through corrosion and pressure checks. Operational measures should limit cooling and hydrate formation, in accordance with ISO 27913.

### 7.1 Limitations

This study had two main limitations. First, the forecasts were performed on a sector model that captures the full target channel within the selected domain but not the full-field dynamic context. The results are therefore most appropriate for scenario screening and pilot-oriented comparison within the modeled area, while field-wide pressure communication, boundary support, and pattern interference may affect the exact magnitude of incremental oil, breakthrough timing, and net CO_2_ storage. Thus, the relative ranking of scenarios is considered more robust than their exact field-scale response.

Second, the model did not include reactive geochemical modeling of CO_2_-brine-rock interactions. Possible effects on mineral reactions, rock properties, injectivity, and long-term trapping were, therefore, not represented explicitly. The reported storage metrics should accordingly be interpreted as engineering estimates under nonreactive assumptions over the forecast period.

Within these limits, the study still provides a useful basis for identifying promising CO_2_ injection and WAG strategies for the target sectors. The conclusions regarding miscible feasibility and the comparative performance of the evaluated scenarios remain valid within the assumptions adopted here, while full-field upscaling and reactive transport assessment should be addressed in future work.
